# Diagnostic and Clinicopathological Utility of High-Molecular-Weight Cytokeratin (HMWCK) and NKX3.1 Immunohistochemistry in Prostatic Adenocarcinoma

**DOI:** 10.7759/cureus.111175

**Published:** 2026-06-19

**Authors:** Ayushi Singh, Zeenat S Imam, Mamta Kumari, Bipin Kumar, Nikhil Ranjan

**Affiliations:** 1 Pathology, Indira Gandhi Institute of Medical Sciences, Patna, IND; 2 Urology, Indira Gandhi Institute of Medical Sciences, Patna, IND

**Keywords:** 34βe12, high-molecular-weight cytokeratin, immunohistochemistry, nkx3.1, prostate adenocarcinoma

## Abstract

Introduction

Basal cell markers and prostate lineage markers are routinely used as diagnostic adjuncts in prostatic epithelial lesions. High-molecular-weight cytokeratin (HMWCK/34βE12) highlights basal cells, whereas NKX3.1 is a prostate-restricted nuclear transcription factor with established diagnostic value in prostatic adenocarcinoma. The present study evaluated the diagnostic expression pattern of HMWCK and NKX3.1 and assessed whether NKX3.1 expression was associated with adverse clinicopathological parameters.

Materials and methods

This prospective observational study included 58 evaluable prostatic epithelial neoplasms received over 24 months at a tertiary care institute. HMWCK and NKX3.1 immunohistochemistry were performed on formalin-fixed paraffin-embedded tissue. NKX3.1 was scored as 0 (0% positive tumor cells), 1 (1-50%), and 2 (51-100%). Statistical analysis included chi-square/Fisher’s exact tests, Mann-Whitney U test, Spearman correlation, and logistic regression. Serum prostate-specific antigen (PSA) values were available for analysis in 42 adenocarcinoma cases.

Results

The final cohort included 57 prostatic adenocarcinomas and one high-grade prostatic intraepithelial neoplasia. Among carcinoma cases, the mean age was 68.09 ± 8.04 years. HMWCK was absent in all adenocarcinoma cases, while NKX3.1 was positive in 55/57 cases (96.5%). NKX3.1 scores were 0 in two cases (3.5%), 1 in 30 cases (52.6%), and 2 in 25 cases (43.9%). Worst Gleason Grade Group showed a weak but significant inverse correlation with NKX3.1 score (Spearman rho = -0.286, p = 0.031). Perineural invasion (PNI) showed the strongest association with NKX3.1 expression; reduced NKX3.1 expression was present in 20/22 PNI-positive tumors compared with 12/35 PNI-negative tumors (p < 0.001; OR 19.17). Serum PSA also correlated inversely with NKX3.1 score among cases with available values (rho = -0.367, p = 0.017), and core involvement percentage correlated inversely with NKX3.1 score (rho = -0.365, p = 0.006).

Conclusions

HMWCK and NKX3.1 showed complementary diagnostic utility in prostatic adenocarcinoma. HMWCK supported invasive adenocarcinoma by demonstrating basal cell loss, while NKX3.1 was retained in most carcinomas as a sensitive prostatic lineage marker. Reduced NKX3.1 expression was associated with higher worst Grade Group, PNI, serum PSA, tumor core involvement, and adverse clinical status. The strongest association was observed between PNI and reduced NKX3.1 expression.

## Introduction

Prostate cancer remains one of the most important malignancies affecting men worldwide and continues to contribute substantially to cancer-related morbidity and mortality [1,2]. The diagnosis and risk stratification of prostatic adenocarcinoma rely on an integrated assessment of clinical features, serum prostate-specific antigen (PSA), imaging, histomorphology, and immunohistochemistry in selected diagnostic settings. Needle core biopsy remains central to clinical decision-making because the Gleason score, grade group, tumor extent, perineural invasion (PNI), and other pathological variables influence treatment planning [2-5].

The diagnosis of prostatic adenocarcinoma is usually straightforward when typical architectural and cytological features are present. However, small atypical acinar proliferations, limited biopsy material, treatment-naive high-grade carcinoma, and differential diagnoses such as urothelial carcinoma may require immunohistochemical support. High-molecular-weight cytokeratin (HMWCK), particularly clone 34βE12, highlights the basal cell layer of benign prostatic glands. Because invasive acinar adenocarcinoma characteristically lacks basal cells, the absence of HMWCK staining in suspicious glands supports malignancy when interpreted in the correct morphological context [6-8].

NKX3.1 is an androgen-regulated homeobox transcription factor located at chromosome 8p21.2. It participates in prostatic epithelial differentiation, androgen receptor signaling, DNA damage response, and tumor-suppressive pathways [9-13]. Immunohistochemically, NKX3.1 is predominantly nuclear and is highly useful as a marker of prostatic origin, particularly in poorly differentiated and metastatic prostatic adenocarcinoma, where PSA and PSAP may be weak, focal, or absent [9,14-17].

Published literature indicates that NKX3.1 has two clinically relevant dimensions. First, it is frequently retained sufficiently to function as a highly sensitive diagnostic prostatic lineage marker, even in high-grade and metastatic conventional adenocarcinoma [14-17]. Second, biological studies have shown that NKX3.1 expression or function may be reduced during prostate carcinogenesis and progression, although the relationship with Gleason grade, PSA, and stage is not uniform across all studies [10-13]. Therefore, diagnostic positivity and biological attenuation should be interpreted as related but distinct concepts.

The present study evaluated HMWCK and NKX3.1 immunohistochemistry in a predominantly adenocarcinoma-based cohort and analyzed the association of NKX3.1 expression with Gleason score, Grade Group, PNI, serum PSA, tumor core involvement, and clinical outcome. The primary endpoint of the study was to evaluate the diagnostic expression pattern of HMWCK and NKX3.1 in prostatic adenocarcinoma. The secondary exploratory endpoints were to assess the association of NKX3.1 expression with adverse clinicopathological parameters, including Gleason score, Grade Group, PNI, serum PSA, tumor core involvement, and clinical outcome.

## Materials and methods

Study design and setting

This hospital-based prospective observational study was conducted in the Department of Pathology in collaboration with the Department of Urology at Indira Gandhi Institute of Medical Sciences (IGIMS), Patna, India. Prostate core biopsy and transurethral resection of prostate (TURP) specimens received over a 24-month period were evaluated. The study was approved by the Institutional Ethics Committee, IGIMS, Patna (approval number 995/IEC/IGIMS/2023), and written informed consent was obtained from all participants.

Study population

The study included 58 evaluable histopathologically confirmed prostatic epithelial neoplasms. The sample size was determined by consecutive case availability rather than by formal a priori statistical estimation. Since this was a hospital-based pathology study, all eligible histopathologically confirmed prostatic epithelial neoplasm cases received during the defined study period were included. For analyses involving Gleason score, Grade Group, PNI, PSA, core involvement, clinical outcome, and NKX3.1 expression, the single HGPIN case was excluded, and the main analysis was restricted to 57 prostatic adenocarcinoma cases.

Inclusion and exclusion criteria

Patients undergoing prostate core biopsy or TURP with a histopathological diagnosis of prostatic epithelial neoplasm were included. Review cases received in the department were eligible when adequate tissue blocks were available for immunohistochemistry. Patients who had received chemotherapy, radiotherapy, or targeted therapy before biopsy, cases of metastatic carcinoma to the prostate from another primary site, non-epithelial prostatic neoplasms, and inadequate biopsies were excluded.

Procedure and data collection

Clinical and pathological data were recorded from departmental records and case proformas. Formalin-fixed paraffin-embedded tissue sections were stained with H&E. Gleason scoring and grade group assignment were performed according to the modified Gleason grading approach used for prostate biopsy reporting. HMWCK (34βE12) and NKX3.1 immunohistochemistry were performed on representative tissue sections. HMWCK was interpreted as cytoplasmic/basal cell staining and recorded as present or absent. NKX3.1 immunostaining was assessed as nuclear tumor cell staining. Based on the assessment approach described by Gurel et al. [15], staining was evaluated visually according to the proportion of positive tumor cell nuclei. NKX3.1 expression was scored as follows: score 0, no tumor cell nuclear staining; score 1, nuclear positivity in 1-50% of tumor cells; and score 2, nuclear positivity in 51-100% of tumor cells. For statistical analysis, scores 0 and 1 were grouped as reduced expression, while score 2 was considered strong expression. This dichotomization was used to separate absent/focal or limited NKX3.1 expression from diffuse expression and to enable comparison with clinicopathological variables, including Gleason score, Grade Group, PNI, PSA, and clinical outcome.

Data analysis

Data analysis was performed using IBM SPSS Statistics for Windows, version 26.0 (released 2018; IBM Corp., Armonk, NY, USA). Descriptive statistics were expressed as frequencies, percentages, means, and SDs. Categorical variables were assessed with chi-square or Fisher’s exact tests. Ordinal and continuous associations were evaluated using Spearman correlation. Mann-Whitney U and Kruskal-Wallis tests were used for non-parametric group comparisons. Binary logistic regression was performed to evaluate predictors of reduced NKX3.1 expression. Receiver operating characteristic (ROC) curve analysis was performed for the prediction of PNI-positive status and adverse clinical outcome. Serum PSA values recorded as greater than 100 ng/mL and greater than 1000 ng/mL were entered as 101 ng/mL and 1001 ng/mL, respectively, for statistical analysis to enable inclusion in correlation models, while unavailable PSA values were excluded from PSA-specific analyses. A p-value less than 0.05 was considered statistically significant.

## Results

Baseline clinicopathological profile

**Figure 1:**
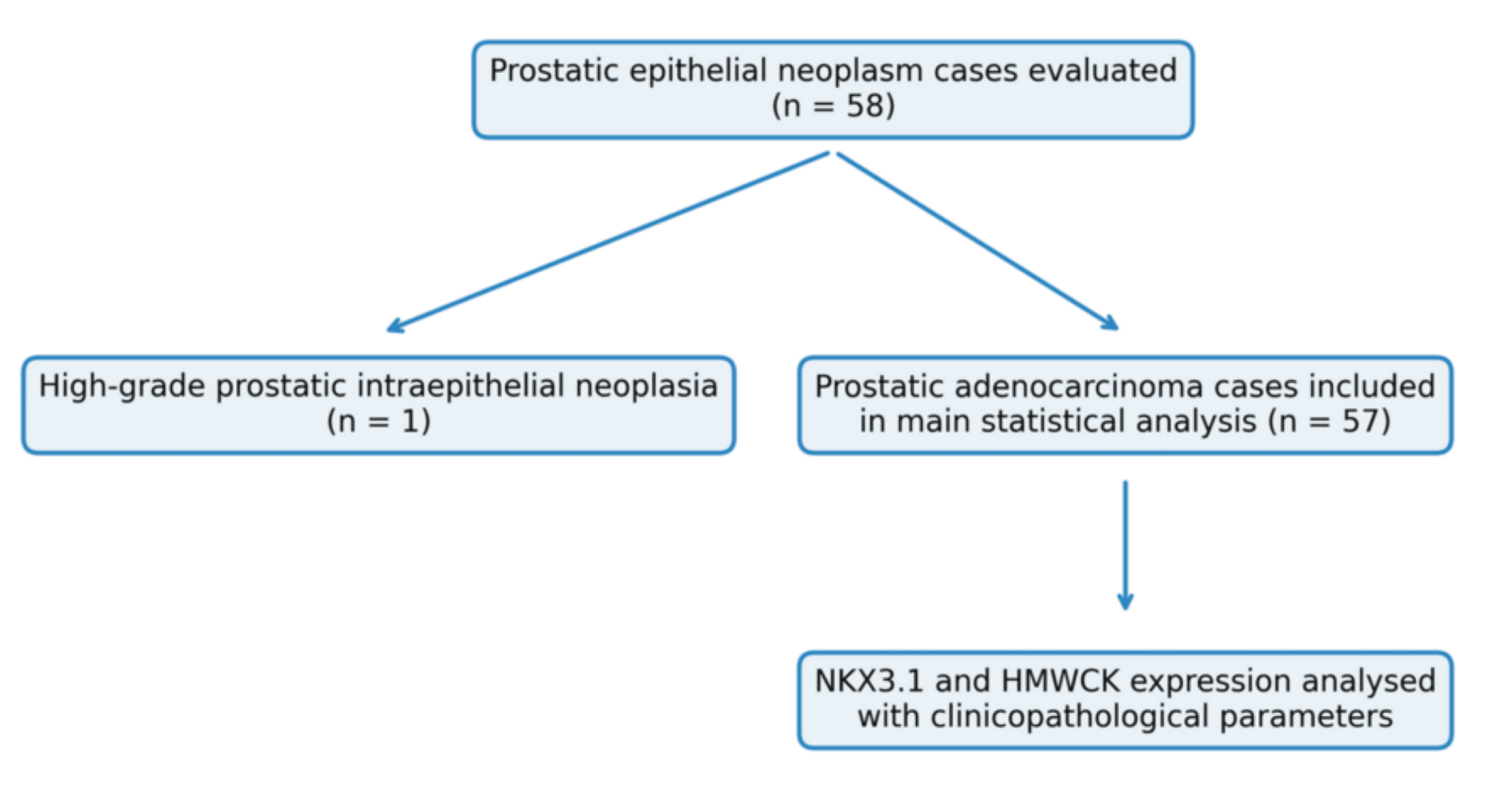
Study flow diagram The figure shows the final evaluable cohort and the carcinoma cases included in the main statistical analysis. HMWCK: high-molecular-weight cytokeratin

**Figure 2:**
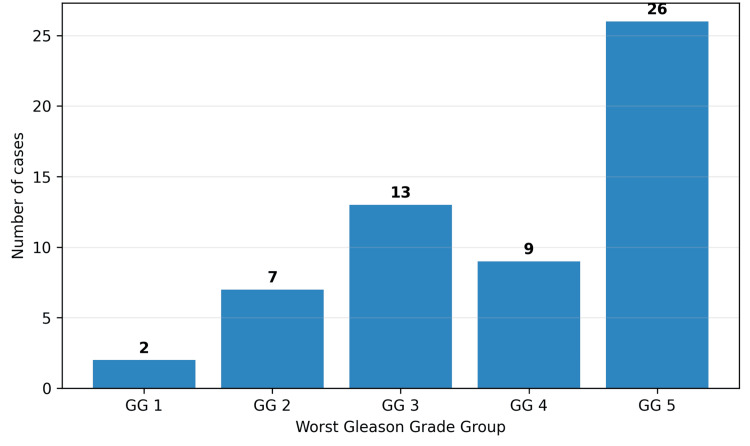
Worst Gleason Grade Group distribution The figure shows high-grade enrichment in the cohort, with Grade Group 5 comprising the largest subgroup.

The final analytical cohort consisted of 57 prostatic adenocarcinoma cases. The mean age was 68.09 ± 8.04 years, with a range of 51-86 years. Core biopsy was the predominant specimen type, accounting for 56/57 cases (98.2%). The cohort was enriched for high tumor burden, with a mean core involvement of 82.39 ± 24.41%. Worst Grade Group 5 was the most frequent category, and Grade Group 4-5 disease was present in 35/57 cases (61.4%). The study flow, baseline characteristics, and grade group distribution are shown in Figure 1, Table 1, Figure 2, and Table 2.

**Table 1:** Baseline clinicopathological characteristics of prostatic adenocarcinoma cases Descriptive statistics are presented as mean ± SD, range, frequency, and percentage, as applicable. PNI: perineural invasion; PSA: prostate-specific antigen; TURP: transurethral resection of prostate

Variable	Statistic or category	Value
Age	Mean ± SD	68.09 ± 8.04 years
	Range	51-86 years
Specimen type	Core biopsy	56 (98.2%)
	TURP	1 (1.8%)
Number of cores	Mean ± SD	10.73 ± 4.07
Cores involved	Mean ± SD	8.64 ± 4.08
Core involvement percentage	Mean ± SD	82.39 ± 24.41%
Worst Gleason score	Mean ± SD	8.09 ± 1.06
Worst Grade Group	Mean ± SD	3.88 ± 1.23
PNI status	Absent	35 (61.4%)
	Present	22 (38.6%)
Serum PSA availability	Cases available for analysis	42/57 cases
Serum PSA	Mean ± SD	108.26 ± 205.32 ng/mL
	Range	0.30-1001.00 ng/mL

**Table 2:** Worst Gleason Grade Group distribution Descriptive frequency analysis was used. Grade Groups 4-5 tumors constituted 61.4% of carcinoma cases, confirming that this was a high-grade-enriched cohort.

Worst Grade Group	Frequency	Percentage
Grade Group 1	2	3.5%
Grade Group 2	7	12.3%
Grade Group 3	13	22.8%
Grade Group 4	9	15.8%
Grade Group 5	26	45.6%
Grade Groups 4-5 combined	35	61.4%

HMWCK and NKX3.1 immunohistochemical expression

**Figure 3:**
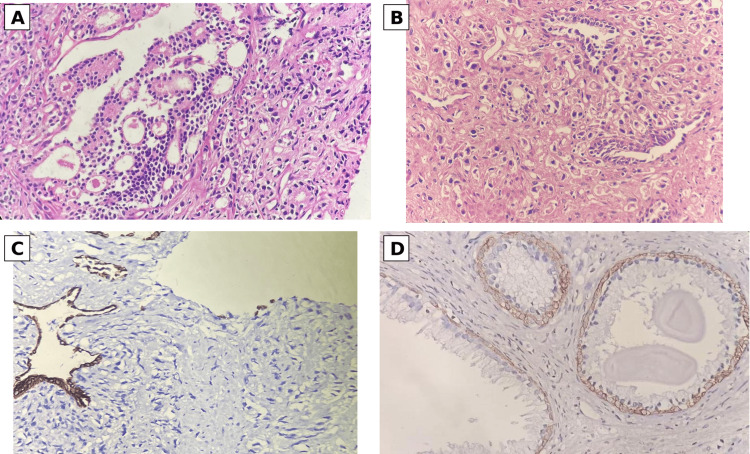
H&E and HMWCK immunohistochemistry in prostatic adenocarcinoma Composite photomicrograph: (A) H&E section showing prostatic adenocarcinoma, predominantly Gleason grade 4 with cribriform and confluent gland pattern (400x); (B) H&E section showing prostatic adenocarcinoma, predominantly Gleason grade 5 with single-cell infiltration pattern (400x); (C) HMWCK immunohistochemistry showing absence of staining in neoplastic glands with retained cytoplasmic positivity in adjacent benign glands (100x); (D) HMWCK positive control showing cytoplasmic positivity in benign prostatic glands (400x). HMWCK: high-molecular-weight cytokeratin

HMWCK was absent in all 57 adenocarcinoma cases, supporting invasive carcinoma through loss of basal cell staining. Since HMWCK was constant within the carcinoma cohort, statistical correlation between HMWCK and NKX3.1 could not be meaningfully assessed among carcinoma cases. NKX3.1 was positive in 55/57 cases (96.5%). Reduced NKX3.1 expression, defined as score 0 or 1, was present in 32/57 cases (56.1%), while strong expression was present in 25/57 cases (43.9%). These findings are summarized in Table 3 and Figure 3.

**Table 3:** Immunohistochemical expression of HMWCK and NKX3.1 in prostatic adenocarcinoma Descriptive frequency analysis was used, and no inferential statistical test was applied because HMWCK was constant within the carcinoma cohort, being absent in all adenocarcinoma cases; therefore, the association between HMWCK and NKX3.1 could not be meaningfully assessed. NKX3.1 showed high diagnostic positivity. HMWCK: high-molecular-weight cytokeratin

Marker/score	Frequency	Percentage	Interpretation
HMWCK absent	57/57	100%	Loss of basal cell staining in invasive carcinoma
HMWCK present	0/57	0%	Not observed in carcinoma cohort
NKX3.1 positive	55/57	96.5%	Retained prostatic lineage nuclear marker
NKX3.1 score 0	2/57	3.5%	Negative
NKX3.1 score 1	30/57	52.6%	Reduced/intermediate expression
NKX3.1 score 2	25/57	43.9%	Strong expression
Reduced NKX3.1 (score 0/1)	32/57	56.1%	Used for dichotomized analysis
Strong NKX3.1 (score 2)	25/57	43.9%	Used for dichotomized analysis

Association of NKX3.1 expression with Grade Group

**Figure 4:**
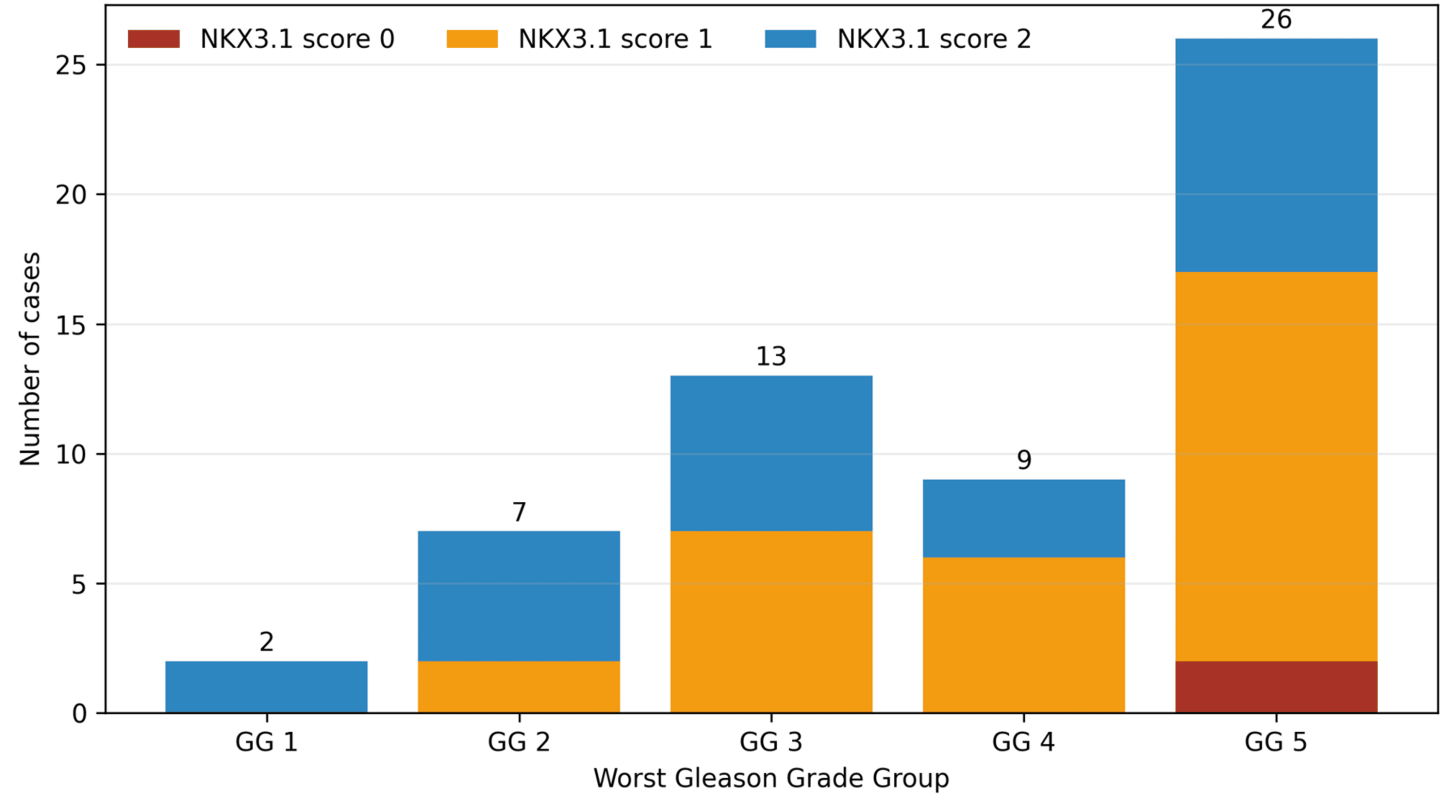
NKX3.1 staining score according to worst Gleason Grade Group The figure illustrates a shift toward reduced NKX3.1 expression in higher grade groups, particularly Grade Groups 4 and 5.

**Figure 5:**
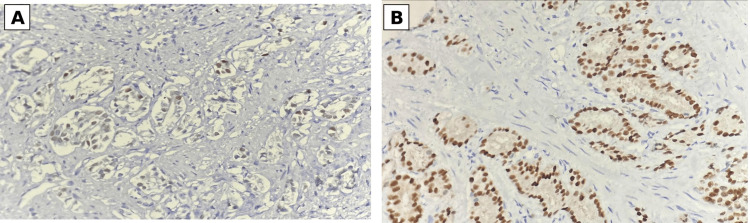
NKX3.1 score 1 and score 2 staining patterns in prostatic adenocarcinoma Composite photomicrograph of NKX3.1 immunohistochemistry: (A) neoplastic glands showing nuclear positivity in up to 50% of tumor cells, corresponding to score 1 (100x); (B) diffuse and strong nuclear positivity in more than 50% of tumor cells, corresponding to score 2 (400x). NKX3.1 was interpreted as nuclear tumor cell staining.

NKX3.1 expression showed a progressive shift toward lower staining scores in higher-grade groups. The full 5 x 3 crosstab did not show a significant Pearson chi-square result (chi-square = 8.074, df = 8, p = 0.426), likely because several cells had small expected counts. However, the linear-by-linear association was significant (p = 0.018), and Spearman correlation demonstrated a weak but statistically significant inverse association between worst Grade Group and NKX3.1 staining score (rho = -0.286, p = 0.031). The distribution of NKX3.1 staining across grade groups is shown in Figure 4 and Table 4, with representative staining patterns shown in Figure 5.

**Table 4:** Worst Grade Group vs NKX3.1 staining score The Pearson chi-square test was used for the full 5 × 3 crosstab between worst Grade Group and NKX3.1 staining score, which was not statistically significant (χ² = 8.074, df = 8, p = 0.426). However, a significant ordinal trend was observed using the linear-by-linear association test (χ² = 5.568, df = 1, p = 0.018), and Spearman correlation demonstrated a significant inverse association between worst Grade Group and NKX3.1 score (rho = −0.286, p = 0.031). Several cells had low expected counts.

Worst Grade Group	NKX3.1 score 0	NKX3.1 score 1	NKX3.1 score 2	Total	Test statistic	p-value
GG 1	0	0	2	2	χ² = 8.074	0.426
GG 2	0	2	5	7		
GG 3	0	7	6	13		
GG 4	0	6	3	9		
GG 5	2	15	9	26		
Total	2	30	25	57		

Association of perineural invasion with NKX3.1 expression

**Figure 6:**
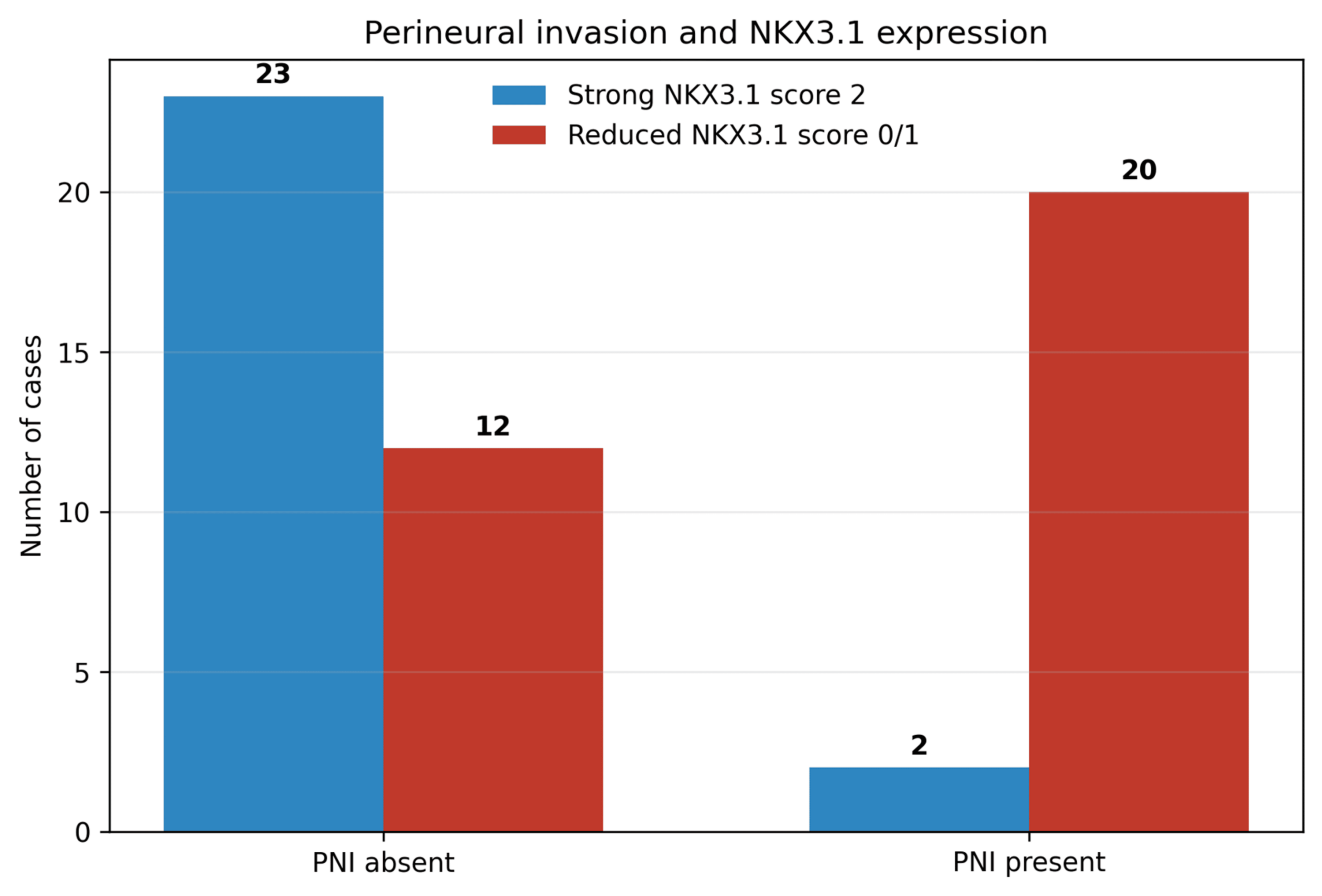
PNI and NKX3.1 expression PNI-positive tumors showed predominantly reduced NKX3.1 expression, whereas PNI-negative tumors more often retained strong NKX3.1 expression. PNI, perineural invasion

**Figure 7:**
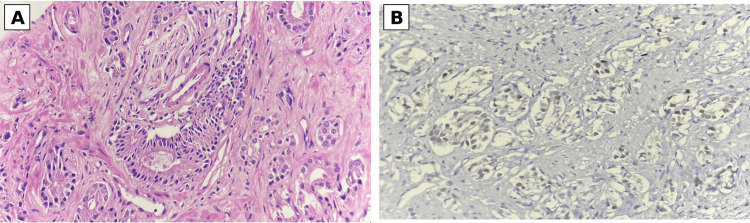
PNI and reduced NKX3.1 expression Composite photomicrograph: (A) H&E section showing prostatic adenocarcinoma, predominantly Gleason grade 4, with perineural invasion (400x); (B) representative NKX3.1 immunohistochemistry showing reduced nuclear positivity in up to 50% of tumor cells, corresponding to score 1 (100x). PNI: perineural invasion

PNI was present in 22/57 adenocarcinoma cases (38.6%). PNI showed the strongest association with NKX3.1 expression. Reduced NKX3.1 expression was present in 20/22 PNI-positive cases (90.9%) compared with 12/35 PNI-negative cases (34.3%). The association between PNI and NKX3.1 staining score was highly significant (chi-square = 17.731, p < 0.001; Cramer V = 0.558). In dichotomized analysis, PNI-positive cases had markedly higher odds of reduced NKX3.1 expression (OR 19.17; Fisher’s exact p < 0.001). These findings are shown in Figure 6, Table 5, and Figure 7.

**Table 5:** PNI status vs NKX3.1 expression The Pearson chi-square test was used to assess the association between PNI status and the three-tier NKX3.1 staining score (χ² = 17.731, df = 2, p < 0.001; Cramer’s V = 0.558). The association between PNI and NKX3.1 staining score was highly significant. Mann-Whitney U testing also showed a significantly lower NKX3.1 score and percentage in PNI-positive tumors (NKX3.1 score: U = 171.000, Z = -3.998, p < 0.001; NKX3.1 percentage: U = 183.000, Z = -3.333, p = 0.001). In dichotomized analysis, PNI-positive cases had markedly higher odds of reduced NKX3.1 expression (OR = 19.17, 95% CI: 3.82-96.12; Fisher’s exact p < 0.001). PNI: perineural invasion

PNI status	NKX3.1 score 0	NKX3.1 score 1	NKX3.1 score 2	Reduced NKX3.1 (score 0/1)	Total	Test statistic	p-value
PNI absent	1	11	23	12	35	χ² = 17.731	<0.001
PNI present	1	19	2	20	22		
Total	2	30	25	32	57		

Association of serum PSA and tumor core involvement with NKX3.1 expression

Serum PSA values were available for analysis in 42 carcinoma cases. Higher serum PSA showed a significant inverse correlation with NKX3.1 staining score (rho = -0.367, p = 0.017), while the association with NKX3.1 percentage showed a borderline inverse trend (rho = -0.289, p = 0.064). Tumor burden assessed by core involvement percentage correlated positively with worst Grade Group (rho = 0.419, p = 0.001) and inversely with the NKX3.1 score (rho = -0.365, p = 0.006). These correlations are summarized in Table 6.

**Table 6:** Key Spearman correlations involving NKX3.1 expression Spearman’s rank correlation was used because several variables were ordinal and/or non-normally distributed. The Spearman rho value is the test statistic for each correlation. Serum PSA correlations were calculated only for cases with available PSA values. PNI: perineural invasion; PSA: prostate-specific antigen

Variables	Spearman rho	Interpretation	Test statistic	p-value
Worst Grade Group vs NKX3.1 score	-0.286	Weakly significant inverse association	rho = -0.286	0.031
Worst Gleason score vs NKX3.1 score	-0.286	Weakly significant inverse association	rho = -0.286	0.031
Worst Grade Group vs NKX3.1 percentage	-0.183	Negative, not significant	rho = -0.183	0.173
Serum PSA vs NKX3.1 score	-0.367	Significant inverse association	rho = -0.367	0.017
Serum PSA vs NKX3.1 percentage	-0.289	Borderline inverse trend	rho = -0.289	0.064
Core involvement percentage vs NKX3.1 score	-0.365	Significant inverse association	rho = -0.365	0.006
Core involvement percentage vs worst Grade Group	0.419	Significant positive association	rho = 0.419	0.001
Core involvement percentage vs PNI	0.346	Significant positive association	rho = 0.346	0.009

Clinical outcome and regression analysis

Clinical outcome categories were heterogeneous, and follow-up duration was short. Symptom-free survival on medical treatment was recorded in 26 cases (45.6%), surgical removal with disease-free status in seven cases (12.3%), death due to disease in 15 cases (26.3%), poor clinical condition on medical treatment in one case (1.8%), and loss to follow-up in eight cases (14.0%). In binary analysis, excluding lost-to-follow-up cases, reduced NKX3.1 expression and PNI were significantly associated with adverse outcomes. The outcome and multivariable regression findings are summarized in Table 7 and Table 8.

**Table 7:** Reduced NKX3.1 expression and PNI in relation to adverse outcomes The Pearson chi-square test was used to compare adverse outcomes with reduced NKX3.1 expression and PNI status. Cases lost to follow-up were excluded from the analysis. Reduced NKX3.1 expression was significantly associated with adverse outcomes (χ² = 11.751, df = 1, p = 0.001; OR = 20.36, 95% CI: 2.40-172.80), as was PNI (χ² = 21.434, df = 1, p < 0.001; OR = 31.50, 95% CI: 5.61-176.90). These findings should be interpreted as exploratory due to short follow-up and heterogeneity in treatment categories. PNI: perineural invasion

Comparison	Favorable outcome	Adverse outcome	Effect estimate	Test statistic	p-value
Strong NKX3.1 score 2	19	1	Reference group	χ² = 11.751	0.001
Reduced NKX3.1 score 0/1	14	15	OR 20.36 for adverse outcome		
PNI absent	27	2	Reference group	χ² = 21.434	<0.001
PNI present	6	14	OR 31.50 for adverse outcome		

**Table 8:** Multivariable logistic regression for reduced NKX3.1 expression Binary logistic regression was used to evaluate predictors of reduced NKX3.1 expression. Wald chi-square values are shown for individual predictors, and the Omnibus chi-square value is reported for the overall model. PNI was the only independent predictor of reduced NKX3.1 expression in the more stable model that preserved sample size, and the clinically interpretable OR is presented for PNI-positive status. -2LL: -2 log likelihood; PNI: perineural invasion

Predictor	Adjusted OR	95% CI	Interpretation	Test statistic	p-value
Age	1	0.92-1.08	Not significant	Wald χ² = 0.000	0.996
Worst Grade Group	0.87	0.46-1.64	Not significant after adjustment	Wald χ² = 0.190	0.663
PNI-positive status	16.13	2.79-90.91	Independent predictor of reduced NKX3.1 expression	Wald χ² = 9.624	0.002
Core involvement percentage	1.03	0.99-1.06	Not significant after adjustment	Wald χ² = 2.526	0.112
Model summary	-2LL 54.801	Nagelkerke R² 0.431	Overall classification 78.6%	Omnibus χ² = 21.685	<0.001

ROC curve analysis

**Figure 8:**
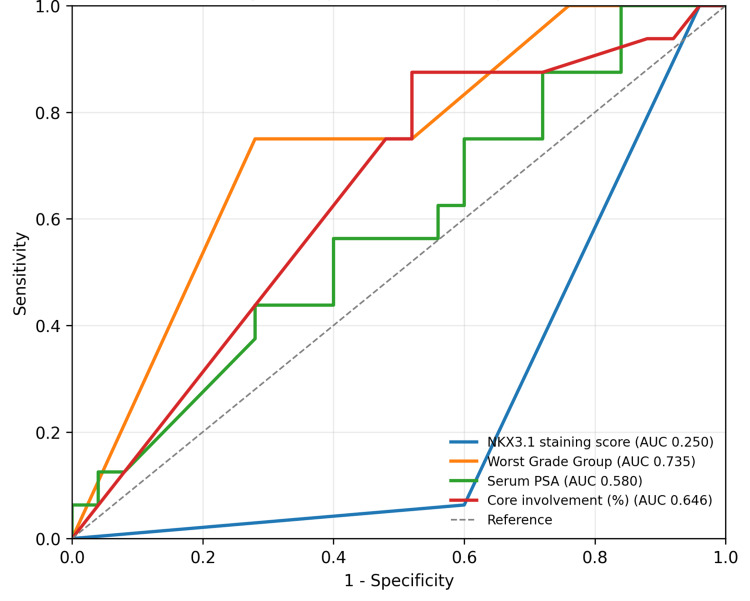
ROC curve for prediction of PNI-positive status NKX3.1 staining score shows an inverse direction because a lower NKX3.1 score was associated with PNI-positive status. AUC: area under the curve; PNI: perineural invasion; PSA: prostate-specific antigen; ROC: receiver operating characteristic

**Figure 9:**
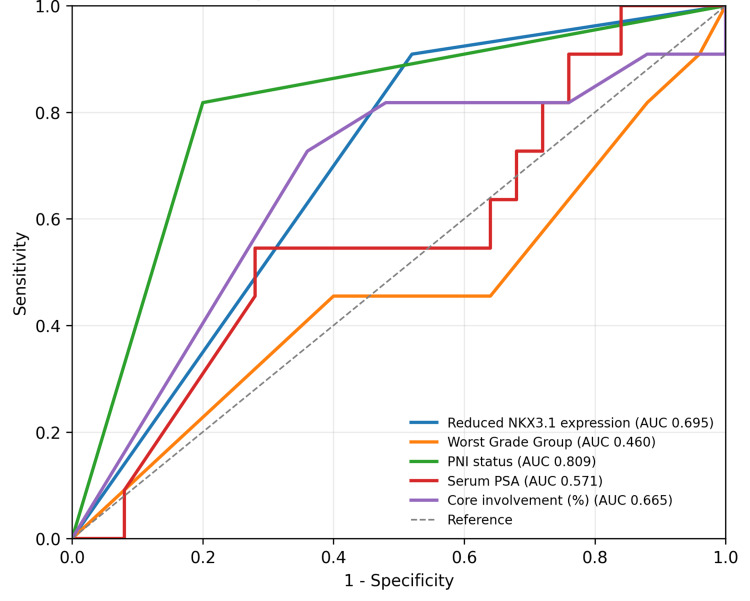
ROC curve for the prediction of adverse outcomes PNI status showed the highest AUC for adverse outcome in this exploratory analysis. AUC: area under the curve; PNI: perineural invasion; PSA: prostate-specific antigen; ROC: receiver operating characteristic

ROC curve analysis was performed as an exploratory analysis. For the prediction of PNI-positive status, worst Grade Group had an area under the curve (AUC) of 0.735 (p = 0.012). NKX3.1 staining score had an AUC of 0.250 (p = 0.008), indicating inverse discriminatory direction because a higher NKX3.1 score was associated with a lower probability of PNI. For adverse outcome, PNI status showed the highest AUC (0.809, p = 0.004), followed by reduced NKX3.1 expression (AUC 0.695, p = 0.066). The ROC curves and AUC values are shown in Figure 8 and Figure 9 and Table 9. Because lower NKX3.1 scores were associated with PNI-positive status, the AUC below 0.5 reflects inverse discriminatory direction rather than absence of biological association.

**Table 9:** ROC analysis ROC curve analysis was used to assess discriminatory performance for PNI-positive status and adverse outcome. The AUC value is shown as the test statistic for each ROC curve variable. NKX3.1 staining score for PNI-positive status had an AUC below 0.5 because a lower staining score, rather than a higher staining score, was associated with PNI-positive status. AUC: area under the curve; PNI: perineural invasion; PSA: prostate-specific antigen; ROC: receiver operating characteristic

Outcome and variable	AUC	SE	95% CI	Test statistic	p-value
PNI-positive status: NKX3.1 staining score	0.25	0.077	0.098-0.402	AUC = 0.250	0.008
PNI-positive status: Worst Grade Group	0.735	0.08	0.579-0.891	AUC = 0.735	0.012
PNI-positive status: Serum PSA	0.58	0.091	0.401-0.759	AUC = 0.580	0.392
PNI-positive status: Core involvement percentage	0.646	0.088	0.474-0.819	AUC = 0.646	0.118
Adverse outcome: Reduced NKX3.1 expression	0.695	0.09	0.518-0.871	AUC = 0.695	0.066
Adverse outcome: Worst Grade Group	0.46	0.112	0.240-0.680	AUC = 0.460	0.706
Adverse outcome: PNI status	0.809	0.083	0.647-0.971	AUC = 0.809	0.004
Adverse outcome: Serum PSA	0.571	0.105	0.365-0.777	AUC = 0.571	0.503
Adverse outcome: Core involvement percentage	0.665	0.105	0.460-0.871	AUC = 0.665	0.118

## Discussion

This study evaluated the diagnostic and clinicopathological utility of HMWCK and NKX3.1 in a tertiary-care cohort predominantly comprising prostatic adenocarcinoma, with one HGPIN case included only in the initial evaluable cohort. The main findings were the following: HMWCK was absent in all adenocarcinoma cases; NKX3.1 was positive in 96.5% of carcinomas; worst Grade Group showed a weak but significant inverse association with NKX3.1 staining score; PNI showed the strongest association with reduced NKX3.1 expression; and serum PSA and core involvement percentage also showed inverse correlations with NKX3.1 score.

The HMWCK findings support the established diagnostic principle that invasive prostatic adenocarcinoma lacks a basal cell layer. ISUP recommendations support the use of basal cell markers, including HMWCK/34βE12 and p63, often in combination with AMACR, for selected morphologically difficult atypical glandular proliferations [6]. Oliai et al. cautioned that rare adenocarcinomas may show basal cell marker reactivity in a basal cell distribution, particularly in low-grade lesions or lesions associated with HGPIN, and therefore emphasized the need for careful morphological correlation [7]. Hasan et al. also demonstrated the usefulness of CK34βE12 with AMACR in resolving suspicious prostatic lesions [8]. In the present cohort, HMWCK was uniformly absent in carcinoma, making it diagnostically useful but unsuitable for carcinoma-only correlation analysis because there was no variability in expression.

The high NKX3.1 positivity rate in the present study is consistent with the literature supporting NKX3.1 as a highly sensitive marker of prostatic origin. Chuang et al. reported NKX3.1 sensitivity of 92.1-94.7% in high-grade prostate adenocarcinoma and 100% specificity against high-grade urothelial carcinoma [9]. Gurel et al. reported 98.6% sensitivity and 99.7% specificity for NKX3.1 in metastatic prostatic adenocarcinoma [15]. Gan et al. and Wang et al. further supported its diagnostic utility in cytology specimens, where NKX3.1 outperformed PSA and PAP/PSAP in detecting metastatic prostatic carcinoma [16,17]. The 96.5% positivity rate in the present study is therefore strongly aligned with published diagnostic literature.

Although NKX3.1 was retained in most tumors, a higher worst Grade Group was associated with a lower NKX3.1 staining score. This is biologically plausible because NKX3.1 is involved in prostate differentiation and tumor-suppressive pathways. Bethel et al. reported decreased NKX3.1 protein expression in focal prostatic atrophy, prostatic intraepithelial neoplasia, and adenocarcinoma, with inverse correlation between carcinoma staining and Gleason grade [11]. Bowen et al. also reported progressive loss of NKX3.1 expression with tumor progression [12].

Griffin et al. summarized contemporary knowledge by noting that the NKX3.1 function is frequently reduced in prostate cancer and that immunohistochemical levels may show a negative correlation with stage and grade [13]. In the evolving framework of precision uro-oncology, such histopathological biomarkers are increasingly interpreted alongside multimodal clinical, imaging, and molecular data to improve risk stratification and personalized decision-making in prostate cancer, as highlighted in recent literature on multimodal precision oncology [18].

The relationship between NKX3.1 and tumor grade is not uniform across all studies. Aslan et al. found significantly lower NKX3.1 staining scores in prostate cancer compared with BPH, but did not find a significant relationship with Gleason score, tumor volume, extraprostatic extension, tumor stage, or serum PSA [10]. Therefore, the present finding should not be interpreted as proof that NKX3.1 is a standalone prognostic marker. Rather, it suggests that reduced NKX3.1 staining may accompany adverse tumor biology in this cohort while NKX3.1 remains diagnostically valuable as a prostatic lineage marker.

The strongest observation in the present study was the inverse association between PNI and NKX3.1 expression. Direct literature examining PNI specifically against NKX3.1 is limited, making this finding exploratory. However, it is indirectly supported by the known adverse significance of PNI. Vargas et al. reported that prostate biopsy specimens with PNI had more tumor-involved cores, higher biopsy Gleason scores, and a greater likelihood of extraprostatic extension [19]. Lee et al. found PNI to be a marker of pathologically advanced disease in localized prostate cancer [20]. More recently, de la Calle et al. reported that PNI in Grade Group 1 prostate cancer on active surveillance was associated with grade reclassification and greater extraprostatic extension at prostatectomy [21]. Ozkaya et al. found that PNI on TRUS-guided biopsy was associated with tumor upgrading and adverse pathological findings [22]. Siech et al. reported that PNI was associated with biochemical recurrence in univariable analysis, although not independently after multivariable adjustment [23]. These findings support interpreting PNI as an adverse-associated histological feature rather than an isolated independent determinant.

The inverse association between serum PSA and NKX3.1 score in this study is plausible but should be considered secondary. Higher PSA may reflect tumor burden, glandular obstruction, inflammation, or advanced disease biology. In contrast, Aslan et al. did not find a significant correlation between NKX3.1 expression and serum PSA [10]. The difference may be related to cohort composition, high-grade enrichment, and the limited number of cases with available PSA values in the present dataset.

Core involvement percentage provided internal pathological consistency. It correlated positively with worst Grade Group and PNI and inversely with the NKX3.1 score. These findings support a coherent pattern in which tumors with greater biopsy burden and adverse histology showed more frequent attenuation of NKX3.1 expression. The outcome and ROC analyses provided clinically relevant exploratory observations, particularly regarding the association of reduced NKX3.1 expression and PNI with adverse clinical status. However, these findings should be interpreted as hypothesis-generating because of the short follow-up duration and heterogeneity of treatment categories.

Limitations and scope for further research

Several limitations of the present study should be acknowledged. First, the sample size was modest, with 57 carcinoma cases in the main analysis, limiting the stability of multivariable models. Second, the cohort was biopsy-enriched and high-grade-enriched, which may limit generalizability to screening-detected low-risk populations. Third, serum PSA values were available for 42 of 57 carcinoma cases, so PSA-related findings should be interpreted as secondary. Fourth, HMWCK was uniformly absent in adenocarcinoma, preventing meaningful correlation analysis of HMWCK within the carcinoma cohort. Fifth, follow-up was short, and outcome categories were heterogeneous; therefore, outcome and ROC analyses should be regarded as exploratory and interpreted cautiously due to sample size constraints. Sixth, serum PSA values reported as greater than 100 ng/mL or greater than 1000 ng/mL were entered as 101 ng/mL and 1001 ng/mL for statistical analysis; however, this approach may introduce minor estimation bias and should be interpreted cautiously. Finally, molecular testing for NKX3.1 deletion, 8p loss, PTEN status, or androgen receptor pathway alterations was not performed. Future larger prospective studies with standardized clinical follow-up and molecular correlation are required to validate whether reduced NKX3.1 expression adds independent prognostic information beyond established clinicopathological parameters.

## Conclusions

HMWCK and NKX3.1 demonstrated complementary roles in prostatic adenocarcinoma. HMWCK was absent in invasive adenocarcinoma, supporting loss of basal cell staining, while NKX3.1 was retained in most carcinoma cases, confirming its diagnostic value as a sensitive nuclear marker of prostatic origin. Beyond diagnosis, reduced NKX3.1 expression showed significant associations with higher worst Grade Group, PNI, serum PSA, tumor core involvement, and adverse clinical status. The strongest association was observed between PNI and reduced NKX3.1 expression. These findings suggest that although NKX3.1 remains a robust prostatic lineage marker, attenuation of NKX3.1 staining may accompany adverse histomorphological and clinical features. Larger studies with standardized follow-up are needed to validate its independent prognostic relevance.
